# GABAergic neurons in nucleus accumbens are correlated to resilience and vulnerability to chronic stress for major depression

**DOI:** 10.18632/oncotarget.16411

**Published:** 2017-03-21

**Authors:** Zhaoming Zhu, Guangyan Wang, Ke Ma, Shan Cui, Jin-Hui Wang

**Affiliations:** ^1^ Qingdao University, School of Pharmacy, Qingdao Shandong, 266021, China; ^2^ State Key Laboratory of Brain and Cognitive Science, Institute of Biophysics, Chinese Academy of Sciences, Beijing, 100101, China; ^3^ University of Chinese Academy of Sciences, Beijing, 100049, China

**Keywords:** depression, resilience, neuron, synapse, nucleus accumbens

## Abstract

**Background:**

Major depression, persistent low mood, is one of common psychiatric diseases. Chronic stressful life is believed to be a major risk factor that leads to dysfunctions of the limbic system. However, a large number of the individuals with experiencing chronic stress do not suffer from major depression, called as resilience. Endogenous mechanisms underlying neuronal invulnerability to chronic stress versus major depression are largely unknown. As GABAergic neurons are vulnerable to chronic stress and their impairments is associated with major depression, we have examined whether the invulnerability of GABAergic neurons in the limbic system is involved in resilience.

**Results:**

GABAergic neurons in the nucleus accumbens from depression-like mice induced by chronic unpredictable mild stress appear the decreases in their GABA release, spiking capability and excitatory input reception, compared with those in resilience mice. The levels of decarboxylase and vesicular GABA transporters decrease in depression-like mice, but not resilience.

**Materials and Methods:**

Mice were treated by chronic unpredictable mild stress for three weeks. Depression-like behaviors or resilience was confirmed by seeing whether their behaviors change significantly in sucrose preference, Y-maze and forced swimming tests. Mice from controls as well as depression and resilience in response to chronic unpredictable mild stress were studied in terms of GABAergic neuron activity in the nucleus accumbens by cell electrophysiology and protein chemistry.

**Conclusions:**

The impairment of GABAergic neurons in the nucleus accumbens is associated with major depression. The invulnerability of GABAergic neurons to chronic stress may be one of cellular mechanisms for the resilience to chronic stress.

## INTRODUCTION

Major depressive disorder is featured by anhedonia, interest loss and low self-esteem. Its major etiology is thought to be chronic stressful environment plus genetic susceptibility [1–8]. The sustained stress to the genetically vulnerable individuals leads to the dysfunctions of monoamine, brain-derived neurotrophic factor and hypothalamus-pituitary-adrenal axis [9–13], which evoke neuron atrophy in brain reward circuits, such as the ventral tegmental area, nucleus accumbens, prefrontal cortex and amygdala, in the depressive patients and stress animals [14–20]. However, most of the individuals do not suffer from major depression after experiencing chronic stress, i.e., resilience to chronic stress [21]. The elucidation of endogenous mechanisms underlying resilience to the chronic stress should shed light on developing therapeutic strategies for major depression. Recent studies indicate the molecular mechanisms related to major depression versus resilience [22–27]. However, cell-specific mechanisms in the brain reward circuits remain to be addressed in terms of the resilience and susceptibility to the chronic stress for major depression [28, 29].

The impairments of GABAergic neurons in the limbic system have been found to be associated with major depression [30–46]. GABAergic neurons are vulnerable to many pathogenic factors [47–51]. It remains to be investigated the hypothesis whether these GABAergic neurons are vulnerable to chronic stress as well as involved in the resilience versus susceptibility to chronic stress for the suffering of major depression. The nucleus accumbens includes cell cluster only for GABAergic neurons in the limbic system and is thought to be one of family members in the brain reward circuit [52, 53]. Its functions are presumably an interface of emotion, motivation, cognition and action as well as involved in the reward feeling from the food taking and drug addiction [54–57]. Its dysfunction may be related to anhedonia and interest loss in major depression [58]. Together these studies, we hypothesize that the functional states of GABAergic neurons in the nucleus accumbens may be correlated to either resilience or susceptibility to chronic stress for major depression.

To the questions above, we propose to examine whether the functional states of GABAergic neurons in the nucleus accumbens are correlated to the resilience and susceptibility to chronic stress for major depression. Chronic unpredictable mild stress (CUMS) is applied to treat the mice for three weeks. The sucrose preference, Y-maze and forced swimming tests are used to examine whether their behaviors change significantly. Mice from CUMS-induced depression and resilience as well as from controls were studied about the functions of their GABAergic neurons in the nucleus accumbens by cellular electrophysiology and protein chemistry. GABAergic neurons in these mice were genetically labeled by green fluorescent protein to confirm cell identity [59]. With these analyses, we expect to reveal cell-specific mechanisms underlying resilience versus susceptibility to chronic stresses for major depression.

## RESULTS

### Chronic unpredictable mild stress to the mice leads to either depression-like behaviors or resilience

**Figure 1:**
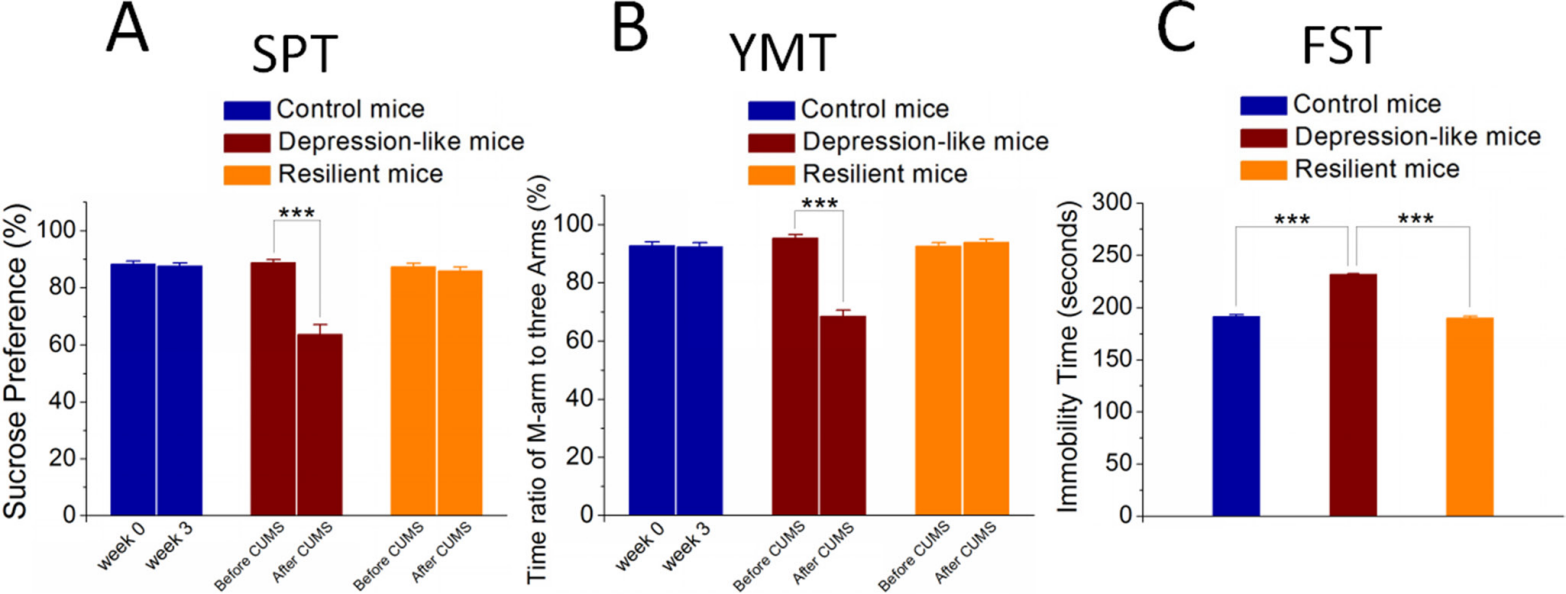
Chronic unpredictable mild stress (CUMS) leads mice to express depression-like behaviors or resilience (**A**) shows the SPT values (%) in the mice from CUMS-induced depression (red bar), CUMS resilience (orange bar) and control group (blue bar). (**B**) illustrates the ratios of stay time in M-arm to stay time in three arms by the YMT in the mice from CUMS-induced depression (red bar), CUMS resilience (orange) and control group (blue). (**C**) illustrate immobile time of staying in the water cylinder by the FST in the mice from CUMS-induced depression (red bar), CUMS resilience (orange) and control group (blue). Three asterisks show *p* < 0.001, in which one-way ANOVA was used for the comparisons among CUMS-induced depression, CUMS resilience and control mice, while paired-*t* test was for the comparisons before and after the CUMS.

The mice were treated by the CUMS or control in three weeks. Their mood states were assessed by sucrose preference test (SPT), Y-maze test (YMT) and forced swimming test (FST). In comparisons with control mice, the mice of receiving the CUMS treatment appear the significant change and no change in all of these three tests, or the significant change in one or two of these tests. These mice are called as major depression, resilience and atypical, respectively. Figure 1 shows statistical analyses for the mice of depression, resilience and control. The SPT values in CUMS-induced depression mice are 63.74 ± 3.37% after the CUMS and 88.78 ± 1.06% before the CUMS (*p* < 0.001, *n* = 15, paired *t-test*; red bars in Figure 1A). The SPT values in the mice of resilience to the CUMS are 85.92 ± 1.3% after the CUMS and 87.33 ± 1.17% before the CUMS (*n* = 11; orange bars). Blue bars in Figure 1A illustrate the SPT values in control mice (*n* = 16). The ratios of stay time in the M-arm to stay time in total arms in CUMS-induced depression mice are 68.59 ± 2.11% after the CUMS and 95.38 ± 1.17% before the CUMS (*p <* 0.001, *n* = 15, paired *t-test*; red bars in Figure 1B), while these values in the mice of resilience to the CUMS are 93.88 ± 1.04% after the CUMS and 92.57 ± 1.21% before the CUMS (*n* = 11, oranges). Blue bars in Figure 1B illustrate the YMT values in control mice (*n* = 16). In addition, the values of immobile time in the FST are 231.4 ± 0.9 seconds in CUMS-induced depression mice (*n* = 15, red bar in Figure 1C), 190.1 ± 1.7 seconds in resilience mice (*n* = 11, orange) and 191.4 ± 1.8 seconds in control mice (*n* = 16, blue; three asterisks, *p <* 0.001, one-way ANOVA). The chronic unpredictable mild stress evokes depression-like behaviors in certain mice versus resilience in others, compared to control mice.

In terms of cellular mechanisms underlying depression-like behavior versus resilience in the mice, we have examined the possibility that the functional states of GABAergic neurons in the nucleus accumbens are involved in the resilience versus vulnerability to chronic stress for major depression. The outputs of GABAergic neurons were studied by analyzing spontaneous inhibitory postsynaptic currents (sIPSC) on GABAergic neurons (i.e., interaction among them) as well as GABA synthesis and reuptake. The intrinsic property of GABAergic neurons was assessed by measuring their input-output curves. The reception of GABAergic neurons to excitatory inputs was evaluated by analyzing spontaneous excitatory postsynaptic currents (sEPSC).

### GABAergic neuron outputs decrease in the nucleus accumbens from depression-like mice, but not resilience

**Figure 2:**
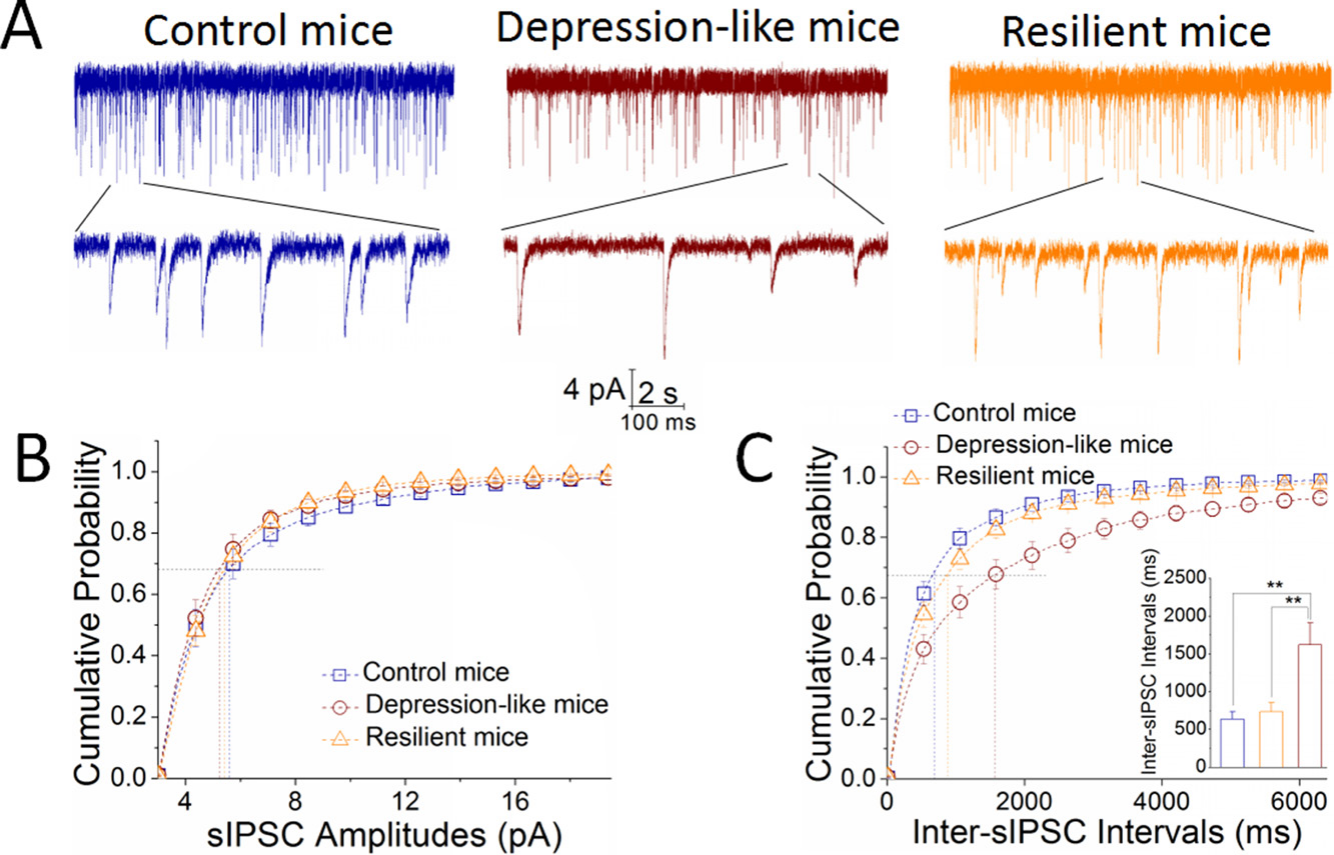
Inhibitory synaptic transmission is downregulated in GABAergic neurons of the nucleus accumbens from CUMS-induced depression mice, but not resilience sIPSCs were recorded under voltage-clamp in the brain slices from control, resilience and depression-like mice in presence of 10 μM CNQX and 40 μM D-AP5. (**A**) Left panel shows sIPSCs from a control mouse (blue traces), middle panel shows sIPSCs from a depression-like mouse (reds) and right panel shows sIPSCs from a resilience mouse (orange). Calibration bars are 4 pA in vertical bar as well as 2 seconds (top traces) and 100 milliseconds (bottoms) in horizontal. (**B**) shows cumulative probability versus sIPSC amplitudes from depression-like mice (red symbols), resilience mice (orange) and control mice (blue). Dash-lines indicate sIPSC amplitudes at cumulative probability to 67% (CP_67_). (**C**) shows cumulative probability versus inter-sIPSC intervals from the depression-like mice (red symbols), resilience mice (orange) and control (blue). Dash-lines indicate sIPSC intervals at CP_67_ in control (blue line; *n* = 12 cells), resilience (orange; *n* = 14 cells) and depression-like mice (red; *n* = 14 cells). The inserted figure shows a comparison of sIPSC intervals at CP_67_ from the mice of CUMS-induced depression (red bar), resilience (orange) and control (blue), in which two asterisks show *p* < 0.01, one-way ANOVA).

By recording sIPSCs on GABAergic neurons in the nucleus accumbens, lower sIPSC frequencies appear to be seen in depression-like mice (red traces), compared to resilience mice (orange) and control mice (blue in Figure 2A). Figure 2B shows cumulative probability versus sIPSC amplitudes in CUMS-induced depression (red symbols), resilience (orange) and control mice (blue). Figure 2C shows cumulative probability versus inter-sIPSC intervals in CUMS-induced depression (red symbols), resilience (orange) and control mice (blue). Inter-sIPSC intervals at 67% cumulative probability are 1625 ± 285 ms in depression-like mice (red bar, *n* = 14 cells from 7 mice), 739 ± 118 ms in resilience mice (orange, *n* = 14 cells from 7 mice) and 638 ± 101 ms in control mice (blue, *n* = 12 cells from 8 mice; *p <* 0.01, one-way ANOVA). There is no difference in sIPSC amplitudes in the three groups of mice. The differences between the CUMS-induced depression mice and the resilience and/or controls may be due to the decreased release of GABA from presynaptic terminals in the nucleus accumbens.

**Figure 3:**
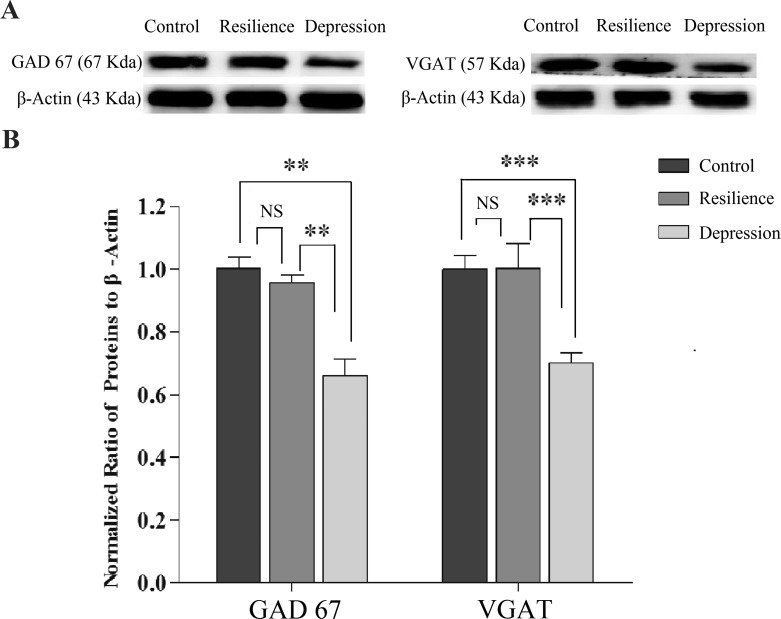
GABA synthesis, uptake and release are impaired in the nucleus accumbens of CUMS-induced depression mice, but not resilience mice The expression and relative quantity of proteins GAD-67 and VGAT were studied by western-blot. (**A)** Left panel shows GAD-67 expressions from the mice of control, CUMS resilience, and CUMS-induced depression, where internal control is done with β-actin. Right panel shows VGAT expressions from the mice of control, CUMS resilience and CUMS-induced depression. (**B)** illustrates the normalized ratios of GAD67 and VGAT to β-actin from control mice (dark-gray bars, *n* = 8), resilience mice (gray, *n* = 4) and CUMS-induced depression mice (light-grays, *n* = 8). The relative ratios for control mice are normalized to be one. An asterisk presents *p* < 0.05, two asterisks denote *p* < 0.01, and three asterisks denote *p* < 0.001 (one-way ANOVA).

In terms of the molecular mechanism underlying the attenuated output ability of GABAergic neurons in the depression-like mice, the proteins related to GABA synthesis and uptake were hypothetically downregulated, which was examined by the western-blot detection of the proteins correlated to these functional processes, such as glutamic acid decarboxylase (GAD-67) and vesicular GABA transporter (VGAT). In other words, the expression of GAD-67 was used to examine GABA synthesis and the level of VGAT was used to merit GABA uptake. Figure 3A–3B shows western-blot analyses about the levels of GAD-67 and VGAT that are harvested from the nucleus accumbens of CUMS-induced depression, resilience and control mice, respectively. The densities of GAD-67 and VGAT bands appear lower in CUMS-induced depression mice, compared with those in resilience and control mice (Figure 3A). Statistical analyses in Figure 3B demonstrate that the levels of GAD-67 and VGAT are significantly lower in the nucleus accumbens from CUMS-induced depression mice than those in resilience and control mice. The consistent results from the studies of electrophysiology and protein chemistry strengthen the reliability of our studies.

### GABAergic neuron excitability decreases in the nucleus accumbens from depression-like mice, but not resilience

**Figure 4:**
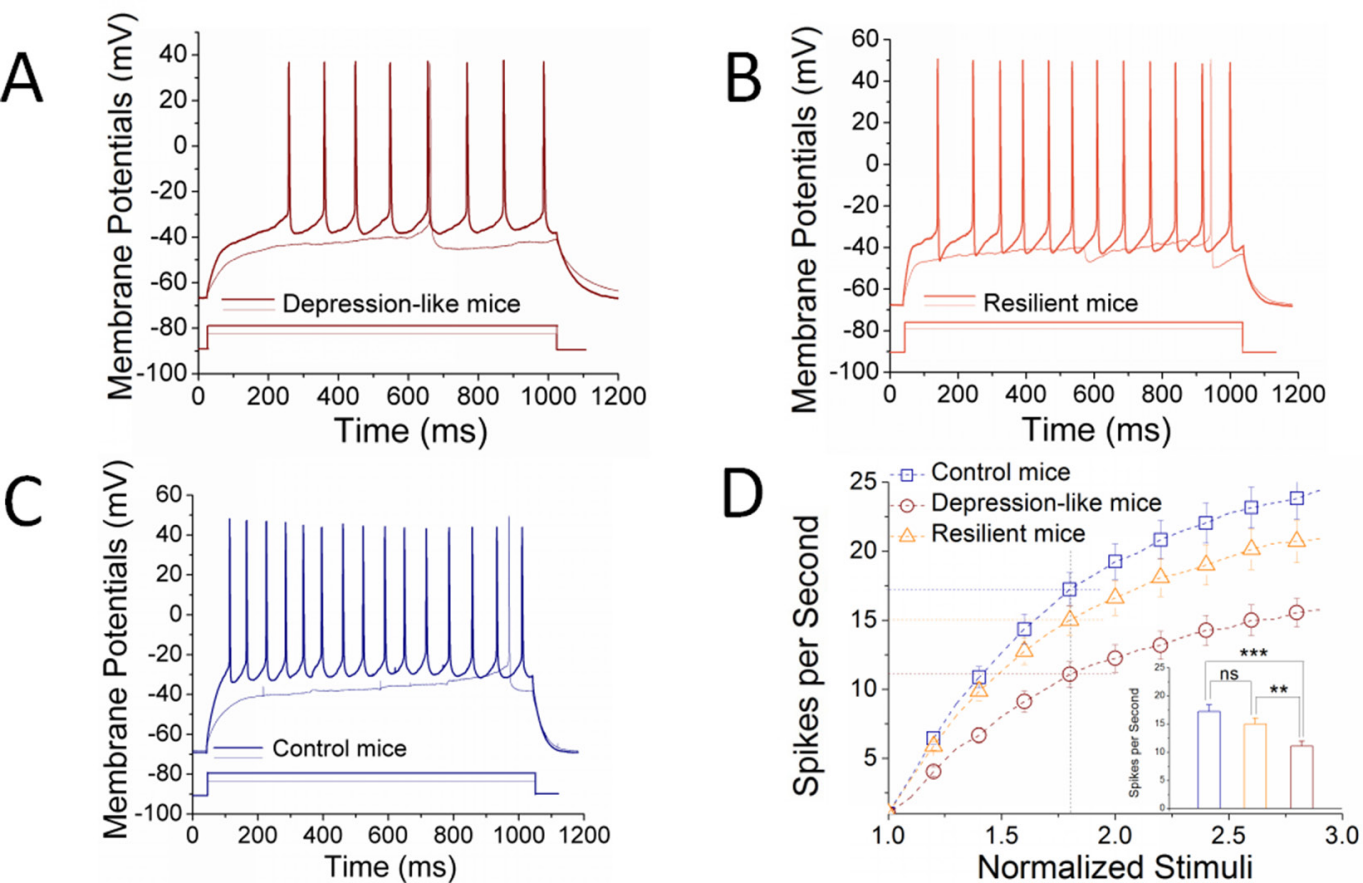
The ability to produce the sequential spikes on GABAergic neurons of the nucleus accumbens decreases in the depression-like mice, but not resilience Sequential spikes induced by various stimulus intensities were recorded on GABAergic neurons in the nucleus accumbens in brain slices under current-clamp. (**A)** illustrates depolarization-induced the sequential spikes on GABAergic neurons from a CUMS-induced depression mouse. (**B)** illustrates depolarization-induced the sequential spikes on GABAergic neurons from a CUMS resilience mouse. (**C)** illustrates depolarization-induced the sequential spikes on GABAergic neurons from a control mouse. (**D)** illustrates spikes per second versus normalized stimuli in GABAergic neurons from the depression-like mice (red symbols, *n* = 25 cells), resilience mice (orange, *n* = 21 cells) and control mice (blue, *n* = 24 cells). Inserted figure shows spikes per second at 1.8 normalized stimuli from depression-like mice (red bar), resilience mice (orange) and control mice (blue), in which two asterisks indicate *p* < 0.01 and three asterisks are *p* < 0.001 (one-way ANOVA).

Figure 4 shows neuronal ability to convert excitatory inputs into spikes. GABAergic neurons in the nucleus accumbens appear lower ability to encode spikes in depression-like mice (Figure 4A), compared to resilience mice (Figure 4B) and control mice (Figure 4C). Figure 4D illustrates spikes per second versus normalized stimuli in the GABAergic neurons from control (blue symbols, *n* = 24 cells from 8 mice), resilience (orange, *n* = 21 cells from 7 mice) and depression (red, *n* = 25 cells from 7 mice). Spikes per second at 1.8 of normalized stimuli are 17.25 ± 1.2 in control mice (blue bar), 15.0 ± 1.1 in resilience mice (orange) and 11.08 ± 0.9 in depression-like mice (red; two asterisks, *p <* 0.01 and three asterisks, *p <* 0.001). It is noteworthy that there is no statistical change between control and resilient mice. The decreased capability to convert excitatory inputs into spikes in GABAergic neurons of the nucleus accumbens is associated with depression-like behavior, but not resilience.

### Excitatory synaptic transmission lowers on nucleus accumbens GABAergic neurons from depression-like mice

**Figure 5:**
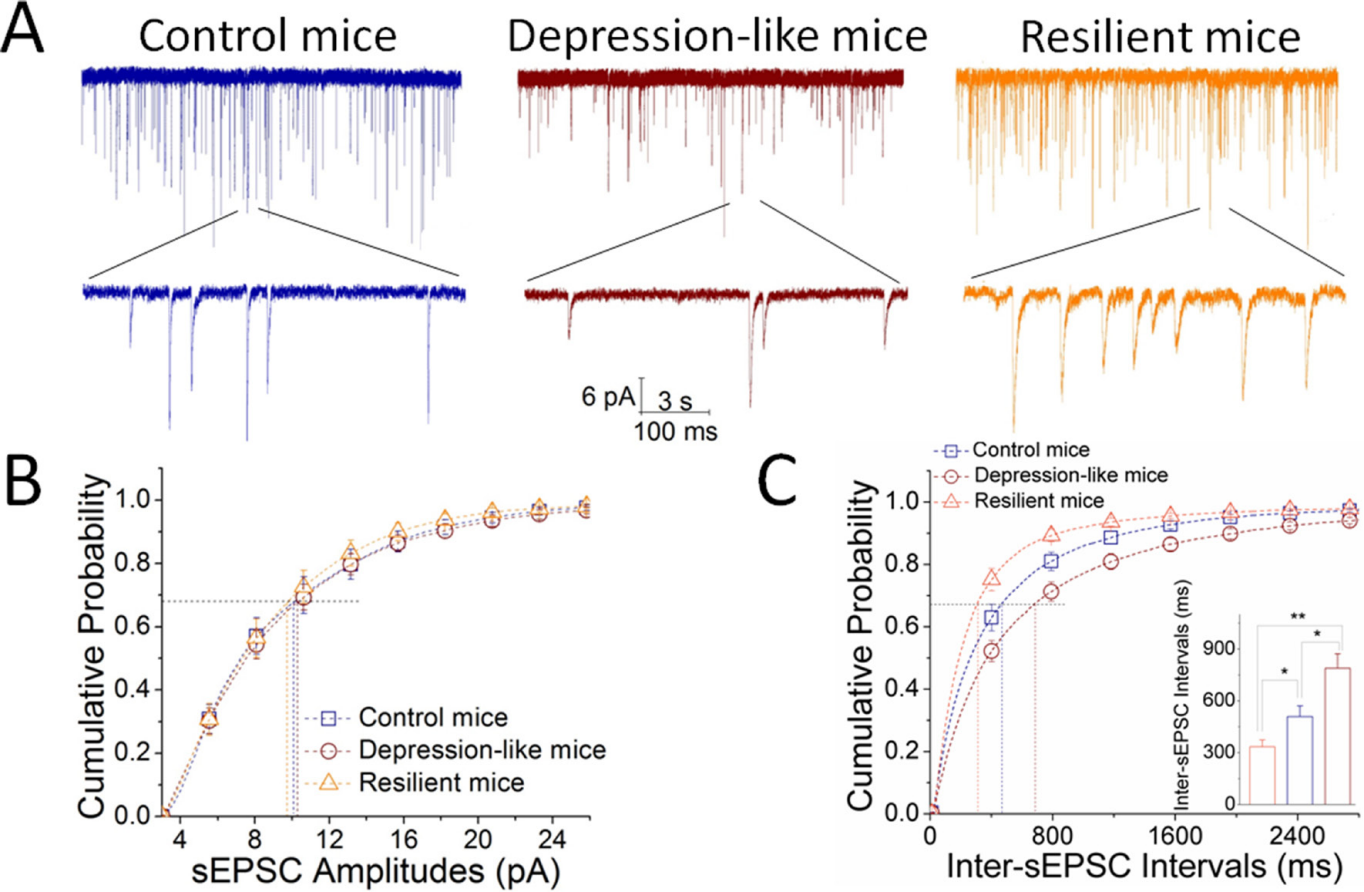
Excitatory synaptic transmission is downregulated in GABAergic neurons of the nucleus accumbens from CUMS-induced depression mice, but not resilience sEPSCs were recorded under voltage-clamp in the brain slices from control, resilience and depression-like mice in presence of 10 mM bicuculline. (**A**) Left panel shows sEPSCs from a control mouse (blue traces), middle panel shows sEPSCs from a depression-like mouse (reds) and right panel shows sEPSCs from a resilience mouse (orange). Calibration bars are 6 pA in vertical bar as well as 3 seconds (top traces) and 100 milliseconds (bottoms) in horizontal. (**B**) shows cumulative probability versus sEPSC amplitudes from depression-like mice (red symbols), resilience mice (orange) and control mice (blue). Dash-lines indicate sEPSC amplitudes at cumulative probability to 67% (CP_67_). (**C**) shows cumulative probability versus inter-sEPSC intervals from the depression-like mice (red symbols), resilience mice (orange) and control mice (blue). Dash-lines indicate sEPSC intervals at CP_67_ in control (blue line; *n* = 23 cells), resilience (orange; *n* = 16 cells) and depression-like mice (red; *n* = 39 cells). The inserted figure shows a comparison of sEPSC interval at CP_67_ from the mice of CUMS-induced depression (red bar), resilience (orange) and control (blue), in which an asterisk shows *p* < 0.05 and two asterisks show *p* < 0.01, one-way ANOVA).

Excitatory synaptic activity was recorded on GABAergic neurons in the nucleus accumbens from control, resilience and depression-like mice (Figure 5). sEPSC frequencies appear higher in resilience mice (orange traces in Figure 5A) than controls (blue), and lower in depression-like mice (red traces) than controls. Figure 5B illustrates cumulative probability versus sEPSC amplitudes in control (blue symbols), resilience (orange) and depression-like mice (red). Figure 5C shows cumulative probability versus inter-sEPSC intervals in the three groups of mice. Inter-sEPSC intervals at 67% cumulative probability are 788 ± 83 ms in depression-like mice (red bar; *n* = 39 cells from 7 mice), 508 ± 62 ms in control mice (blue; *n* = 23 cells from 8 mice) and 333 ± 40 ms in resilience mice (orange; *n* = 16 cells from 7 mice; an asterisk, *p <* 0.05 and two asterisks, *p <* 0.01, one-way ANOVA). There is no difference in sEPSC amplitudes in the three groups of mice. CUMS-induced depression may be caused by the decreased reception of excitatory inputs in GABAergic neurons of the nucleus accumbens, but the resilience may be led by the increased reception.

## DISCUSSION

**Figure 6:**
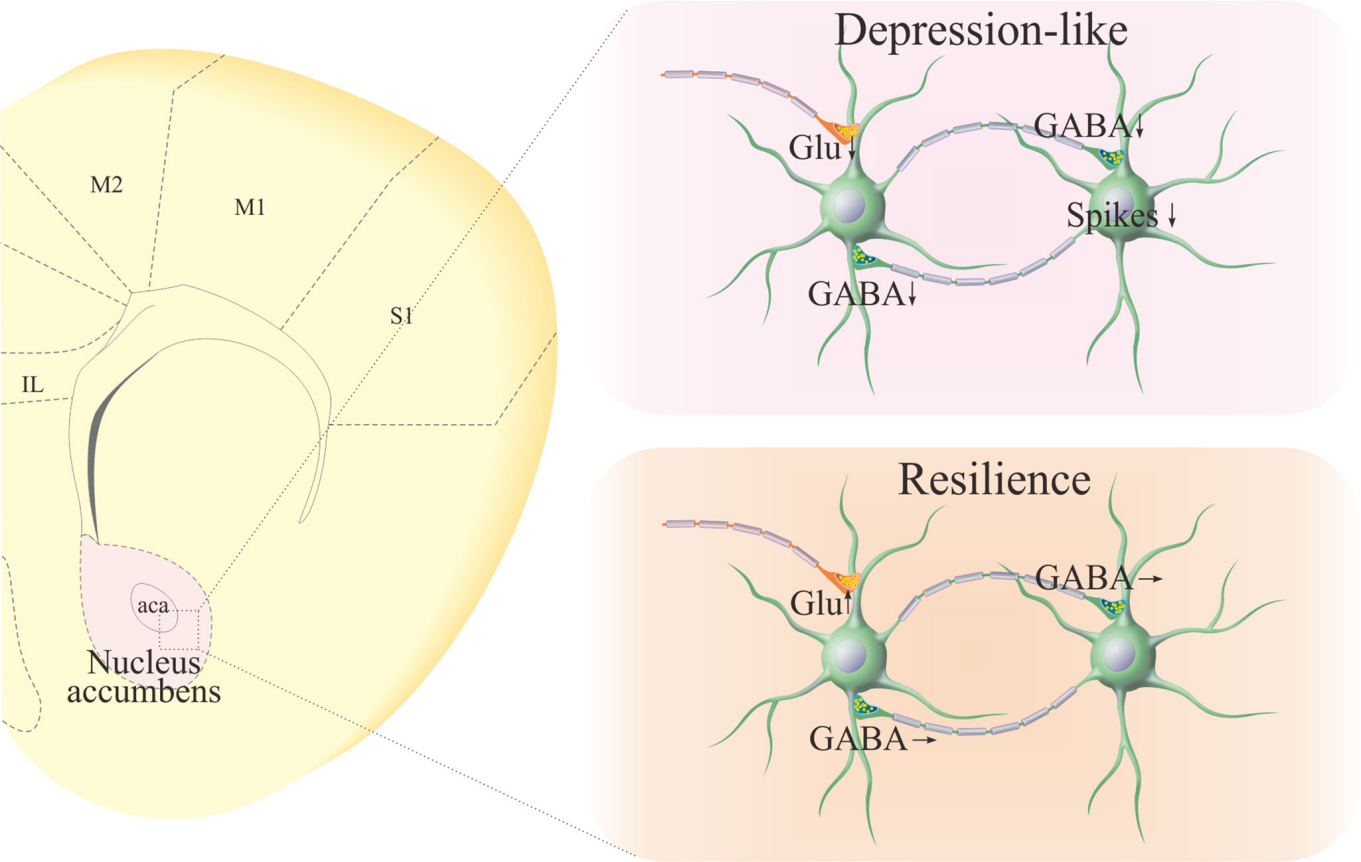
Pathological changes at GABAergic neurons in the nucleus accumbens of CUMS-induced depression mice, compared with those in CUMS-resilience mice Left panel shows the coronal section of the mouse brain including nucleus accumbens. Right-top panel shows the functional downregulation of GABAergic neurons, such as GABA release, neuronal excitability and excitatory input reception. Right-bottom panel illustrates the functional state of GABAergic neurons in the nucleus accumbens of CUMS-resilience mice.

Different to control mice, GABAergic neurons in the nucleus accumbens from CUMS-induced depression mice are featured as the decreases in inhibitory synapse outputs (Figures 2, 3), excitability (Figure 4) and excitatory synapse reception (Figure 5). As the nucleus accumbens includes GABAergic neurons only, all of these attenuated changes in the subcellular compartments of GABAergic neurons lead to the dysfunction of the nucleus accumbens during chronic stress, inducing major depressive disorder. This suggestion is supported the fact that the function of GABAergic neurons in the nucleus accumbens is normal or even upregulated in resilience mice (Figures 2, 3, 4, 5). Thus, the functional state of GABAergic neurons in the nucleus accumbens is correlated to resilience versus vulnerability to chronic stress for major depression (Figure 6).

The nucleus accumbens is thought to be a family member in brain reward circuits [52, 53]. It receives synapse innervations from ventral tegmental area, prefrontal cortex and amygdala. Its physiological roles are presumably the interfaces of various psychological processes, such as emotion, motivation, cognition and action, as well as are involved in the reward feeling from the food taking and drug addiction [54–57]. Its dysfunction may be correlated to anhedonia, interest loss and low motivation seen in major depression [58]. Together these data, we suggest that the induction of major depression may be due to lack of rewards that leads to the poor activation of the brain reward circuits, which is being tested.

Despite the assumption that the chronic stress is a main etiology of major depressive disorder [1–8], most of the individuals after experiencing chronic stress do not suffer from major depression, i.e., resilience to chronic stress [21]. There may be endogenous anti-depression mechanisms for the resilience to chronic stress, and most of the individuals possess a high threshold in response to chronic stress. The elucidation of endogenous mechanisms underlying the resilience to chronic stress should shed light on developing therapeutic strategies for major depression. Recent studies show certain molecules related to major depression versus resilience [22–27]. Here, we present cell-specific mechanism for the resilience to chronic stress that the intact function of GABAergic neurons in the nucleus accumbens is related to the resilience. Together our study with the data that the GABAergic neurons are vulnerable to pathological factors [47–51, 60–63], a worth to be the expected strategy for the treatment of major depression is using the molecular manipulation to shift GABAergic neurons from susceptibility toward resilience to chronic stress.

The ventral tegmental area, nucleus accumbens, amygdala and medial prefrontal cortex are accounted into the brain reward circuit [64–66]. These structures contain many GABAergic neurons, especially the core area of amygdala and the nucleus accumbens mainly include the cluster of GABAergic neurons. The impairment of GABAergic neurons in the limbic system is associated with major depression [30–32, 34–46]. Whether the intact function of the GABAergic neurons in these regions beyond the nucleus accumbens is also associated with resilience to chronic stress remains to be examined in order to get a general view that the function state of GABAergic neurons in the brain is correlated with the resilience versus susceptibility to chronic stress for major depression.

A few of points are worth to be stated. 1) The frequencies of sEPSCs and sIPSCs on GABAergic neurons from the nucleus accumbens decrease in CUMS-induced depression mice (Figures 2 and 5), indicating the changes of presynaptic transmitter release, but not postsynaptic receptors. On the other hand, the excitability of GABAergic neurons from the nucleus accumbens decreases in CUMS-induced depression mice (Figure 4). This datum indicates that CUMS-activated signaling pathways downregulate voltage-gated ion channels (such as sodium, potassium and calcium channels) that set up neuronal excitability, but not postsynaptic ligand-gated ion channels. The mechanisms underlying this differential regulation to different subcellular compartments remain to be addressed. 2) The decrease of sIPSC frequency indicates the attenuated GABA release probability (Figure 2), which is consistent with the data about the attenuated GAD expression for GABA synthesis (Figure 3). In fact, the decreased sIPSC frequency also indicate the lowered density of GABAergic axonal innervations, which remains to be examined. 3) The lowered GABAergic input leads to disinhibition on GABAergic neurons (Figure 2), why does their excitability still decrease (Figure 4)? In fact, the excitatory axon projections from the mediate prefrontal cortex to the nucleus accumbens and synaptic transmission lower in CUMS-induced depression mice (Figure 5 and unpublished data). Their imbalances lead to the dysfunction of GABAergic neurons. On the other hand, the neuronal excitability is set up by postsynaptic voltage-gated ion channels. These voltage-gated ion channels versus ligand-gated ion channels may be differentially regulated by signaling pathways activated by the chronic stress. 4) GABAergic neurons are widely distributed in the shell and core of the nucleus accumbens. Here, we pay attention to investigate the vulnerability versus resilience of these GABAergic neurons in response to the chronic stress. We do not focus on the changes of GABAergic neurons in these sub-regions, since GABAergic neurons in different brain regions are vulnerable to the pathological factors [47–51, 60–63]. The sub-regional changes are worth to be investigated in the future. 5) There is an increased excitatory synaptic input on GABAergic neurons from resilience mice (Figure 5D), which may counterbalance the CUMS-induced change of neuronal excitability (Figure 4) to prevent the occurrence of major depression. The molecular mechanism is being studied.

## MATERIALS AND METHODS

All experiments were done in accordance with the guidelines and regulations by Administration Office of Laboratory Animals at Beijing China. All experimental protocols were approved by Institutional Animal Care Unit Committee in Administration Office of Laboratory Animals at Beijing China (B10831).

### The mouse model of major depressive disorder induced by chronic unpredictable mild stress

In order to examine neuron-specific mechanisms underlying resilience versus susceptibility to chronic stress, we applied C57 GAD-GFP mice whose GABAergic neurons were genetically labeled by green fluorescent protein (GFP) [59]. Male mice were used starting at postnatal day 21. In week one for their adaptation to the experiments, their body weight, locomotion, sucrose preference and Y-maze tests were measured to collect self-control data. The mice of showing consistent value in these measurements at postnatal day 28 were separated into two groups, chronic unpredictable mild stress (CUMS) and control, in order to reduce the variations among these mice. The control mice lived without the following stresses. The use of juvenile mice to examine the occurrence of major depression versus resilience is based on a fact that young individuals have high prevalence to suffer from major depression in response to chronic stress [46].

According to depression risk factors, such as weaknesses in cognitive function, emotional regulation, social interaction skill, circadian and stress response [21], we applied the CUMS to the mice in the following principle. The mice lived in stress environments, made efforts to challenge these stressful conditions and experienced defeat outcomes, which drove them to feel cognitive and emotional inabilities, and in turn to fall into anhedonia and low self-esteem. The protocols for the mice experiencing the CUMS included social isolation, tilted cage, empty cage, damp sawdust cage, restraint space, white noise, strobe light and circadian disturbance [37, 46, 67]. Except for social isolations, these conditions were randomly selected to treat the mice in the manners of their separations or combinations every day. These treatments were applied about 1~14 hours in durations and 1~12 hours in intervals. The durations and intervals were unpredictable to these mice (Table 1 in [46]). This CUMS was sustained for three weeks until some mice expressed anhedonia and low self-esteem. We did not use extreme stress in a single pattern, such as learned hopelessness, electrical shock, social defeat and tail clamp, since these protocols might induce the outcome similar to anxiety, such as posttraumatic stressful disorder [46].

**Table 1:** CUMS-induced behavioral changes in the mice

	Number	Percentage (%)
Depression-like mice	15	28.8%
Resilient mice	11	21.2%
Atypical mice	26	50%
Total CUMS-treated mice	52	100%

Whether the CUMS-treated mice in three weeks fell into anhedonia and low self-esteem was tested in day 29~31. The sucrose preference test (SPT) was used to evaluate anhedonia, the Y-maze test (YMT) were used to assess the loss of interest to their partners and the forced swimming test (FST) was used to estimate their self-esteem [16, 46, 68–71]. The SPT was done by 1% sucrose water versus water for four hours, whose value was presented as the ratio of the ingested sucrose water to the ingested sucrose water plus pure water. The YMT was performed by monitoring mouse staying in a special arm and other two arms for 2 minutes. The end of this special arm included a female mouse (named as M-arm). M-arm stay time was presented by the ratio of stay time in M-arm to that in three arms. The FST was conducted by recording immobile time in a water cylinder (10 centimeters in diameters and 19 centimeters in water depth at 25 ± 1°C). In the quantification of the FST, immobile time was measured. In these tests, the SPT was given once a week, the YMT was given before and after the CUMS, and the FST was given one time after the CUMS. Before the SPT, the mice in groups of CUMS and control were deprived from food and water for 3 hours to drive their intension of drinking water. In the YMT, these arms were cleaned by 70% ethanol and then water after each test to reduce the effect of odor on the test. Carefulness in these tests was taken by performing them in a quiet room, no addition stresses, same circadian circle for all mice and their adaptation in the test environment.

Depression-like behaviors were accepted if the mice in the CUMS group showed the decreases in sucrose preference (twice at ends of week two and three) and M-maze stay time, as well as the increase in immobile time, compared to these values during their self-control period (week one for adaption) and in control group mice. These measurements in each of mice would be considered as significant change if the values of the SPT and YMT reduced above 10% of its self-control values and the value of the FST increased above 10% values from control group mice. These standards set up based on the averaged values in our previous studies [37, 46]. The mice with significant changes in all of these three tests were defined as CUMS-induced depression-like mice or depression-like mice, and those with no changes in these three tests were named as resilience mice. As showed in Table 1, CUMS-treated mice in 3 weeks met this criterion about 28% (i.e., their vulnerability to chronic stress), and 22% of them were resilience (i.e., their invulnerability to chronic stress). These depression and resilience mice were used for the study of electrophysiology. As 30% of CUMS-treated mice met the depression criteria and all CUMS-treated mice did not show a change of the SPT at the end of week one, stressful situations in our study were thought to be mild stress. The mice that met the significant changes in one and/or two measurements are atypical in major depression, called as atypical depression.

### Brain slices and neurons

To have more health brain cells for whole-cell recordings, we prepared the brain slices including nucleus accumbens by the following procedures. The mice were anesthetized by isoflurane inhaling, and were infused by the artificial cerebrospinal fluid (ACSF) and oxygenated (95%O_2_ and 5% CO_2_) at 4°C into their left ventricles until the bodies became cold, in which the concentrations (mM) of the chemicals were 124 NaCl, 3 KCl, 1.2 NaH_2_PO_4_, 26 NaHCO_3_, 0.5 CaCl_2_, 4 MgSO_4_, 10 dextrose and 220 sucrose at pH 7.35. The mouse heads were immediately decapitated by guillotine and placed into this cold oxygenated ACSF with the brain isolation. The brain slices (300 μm) in coronal direction were cut by Vibratome in this cold oxygenated ACSF. They were held in another oxygenated ACSF (124 NaCl, 3 KCl, 1.2 NaH_2_PO_4_, 26 NaHCO_3_, 2 CaCl_2_, 2 MgSO_4_, 10 dextrose, and 5 HEPES, pH 7.35) at 25°C for 2 hours. Each slice was placed into a submersion chamber (Warner RC-26G) that was perfused by the oxygenated ACSF at 31°C for the electrophysiological recordings [72–74]. The chemical reagents were from Sigma.

Whole-cell recording was done on the GFP-labeled GABAergic neurons in the shell or core of the nucleus accumbens under DIC-fluorescent microscope (Nikon FN-E600, Tokyo, Japan). The wavelength at 488 nm excited the fluorescence of GFP-labeled neurons. GABAergic neurons expressed fast spikes with less adaptation in their amplitude and frequency, the typical properties for the interneurons [75–78].

### Whole-cell recording and neuronal functions

The neurons were recorded by MultiClamp-700B amplifier under voltage-clamp for their synaptic activity and the current-clamp for their intrinsic property. Electrical signals were inputted to pClamp-10 (Axon Instrument Inc.) for data acquisition and analysis. An output bandwidth of the amplifier was set at 3 kHz. The pipette solution of recording excitatory events included (mM) 150 K-gluconate, 5 NaCl, 5 HEPES, 0.4 EGTA, 4 Mg-ATP, 0.5 Tris-GTP, and 5 phosphocreatine (pH 7.35; [79–81]. The solution for studying inhibitory synapses contained (mM) 130 K-gluconate, 20 KCl, 5 NaCl, 5 HEPES, 0.5 EGTA, 4 Mg-ATP, 0.5 Tris–GTP and 5 phosphocreatine [82, 83]. These pipette solutions were freshly made and filtered (0.1 μm). The osmolarity was 295~305 mOsmol and pipette resistance was 5~6 MΩ.

The functions of GABAergic neurons were assessed including their active intrinsic properties and inhibitory outputs [50, 84, 85]. Inhibitory outputs were assessed by recording spontaneous inhibitory postsynaptic currents (sIPSC) on GABAergic neurons in the presence of 10 mM 6-Cyano-7-nitroquinoxaline-2,3-(1H,4H)-dione (CNQX) and 40 μM D-amino-5-phosphonovanolenic acid (D-AP5) in the ACSF to block the ionotropic glutamatergic receptors. 10 μM bicuculline was washed onto the slices at the end of experiments to block sIPSCs and test whether synaptic responses were mediated by GABA_A_R. The pipette solution with a high concentration of chloride ions makes reversal potential to be -42 mV. sIPSCs are inward currents when membrane potential is held at -65 mV [83, 86].

The recording of spontaneous postsynaptic currents, instead of evoked synaptic currents, is based on the following reasons. sEPSC and sIPSC amplitudes represent the responsiveness and the densities of postsynaptic receptors. The frequencies imply the probability of transmitter release from an axon terminal and the number of presynaptic axons innervated on the recorded neurons [87, 88]. Such parameters can be used to analyze presynaptic and postsynaptic mechanisms. The evoked postsynaptic currents cannot separate these mechanisms. We did not use TTX into the ACSF to record miniature postsynaptic currents since we had to record neuronal excitability. Synaptic events in our recording are presumably miniature postsynaptic currents. This point is granted by a single peak of postsynaptic currents in our study.

Action potentials at GABAergic neurons in the nucleus accumbens were induced by injecting depolarization pulse. Their excitability was assessed by input-outputs (spikes versus normalized stimuli) when various stimuli were given [89], in which stimulus intensities were step-increasing by 10% normalized stimulations. As the excitability of different neurons was variable so that step-increased depolarization pulses were given based on their normalization. The base value of stimulus intensity for this normalization at each cell was the threshold intensity of depolarization pulse (1000 ms in duration) to evoke a single spike [89]. We did not measure a rheobase to show cellular excitability, since this strength-duration relationship was used to assess the capability to fire single spike. We measured the ability of firing sequential spikes [78].

Data were analyzed if the recorded neurons had the resting membrane potentials negatively more than -60 mV, and action potential amplitudes more than 90 mV. The criteria for accepting each experiment also included less than 5% changes in resting membrane potential, spike magnitude, and input resistance throughout each recording. The series and input resistances in all neurons were monitored by injecting hyperpolarization pulses (5 mV/50 ms), and calculated by voltage pulses versus instantaneous and steady-state currents.

### Western-blot to quantify proteins

The tissues isolated from the nucleus accumbens in each sample of the depression-like mice, resilience mice and control mice were gently washed three times in ice-cold PBS and placed in 1 ml of RIPA Lysate buffer with PMSF (Beyotime Biotechnology, China) for their homogenizations. Homogenized tissues were removed into a new EP tube (1.5 ml), kept at 4°C in the refrigerator for 30 minutes, and centrifuged at 12000 g/min for 15 minutes. The total protein concentration was measured by using protein assay based on the manufacturer's instruction in supernatant liquid. Fifty micrograms of total proteins per sample was resolved in 10% sodium dodecyl sulphate polyacrylamide gel electrophoresis (SDS-PAGE). After their separations, proteins were electrically transferred onto nitrocellulose membranes. These membranes were incubated with blocking solution (1×TBS; 0.1% Tween 20; 5% non-fat milk) at room temperature (25°C) for 2 hours, and then incubated overnight with primary antibodies (1:2000 in dilution) of GAD-67 (ab26116, Abcam, Cambridge UK), VGAT (A3129, ABclonal Technology, Wuhan China), or with primary antibody (1:1000 in dilution) of β-actin (AC004, ABclonal Technology) in 5% milk in TBST. After incubations with their corresponding secondary antibodies conjugated with peroxidase (Beyotime Biotechnology, China), the proteins were visualized by using the enhanced chemiluminescence ECL Plus immunoblotting detection system (Climx Science Instruments Co. Ltd, China). The bands corresponding to the expected sizes were selected on a computerized scanner, and the pixel density in each band was determined by this computer after the background correction for relative quantization. The optical densities of each band relative to measured values of β-actin bands were determined using Image-J software.

### Statistical analyses

The data of behavioral tests, electrophysiological recordings and protein chemistry are presented as mean ± SEM. Paired *t-test* was used in the comparisons of experimental data before and after the CUMS in each of the mice. One-way ANOVA was used to make statistical comparisons in neuronal activity and western-blot among control, resilience and depression-like mice.
